# What Should They Publish about Their Work and Needs?

**Published:** 1915-12-25

**Authors:** W. J. Morton

**Affiliations:** The Mount Vernon Hospital for Consumption and Diseases of the Chest, Fitzroy Square, London, W.


### OUR VOLUNTARY HOSPITALS & INSTITUTIONS.

### What should they Publish about their Work and Needs?

*To the Editor of* The Hospital.

Sir,—" Assistant Secretary " says that he does not remember receiving a single inquiry from the outside public " as to even larger questions of management." I imagine that his experience must be unique in this respect. I can only say that I have received many such inquiries, which have often led to the discussion of points of detail, with persons who had hitherto shown no interest in the hospital.

I am glad to be able to add that in many cases the inquiry has led to further interest.—Yours faithfully,

W. J. Morton.

The Mount Vernon Hospital for Consumption and Diseases of the Chest,
Fitzroy Square, London, W.,
December 20, 1915.

